# A review of the use of controlled multiple imputation in randomised controlled trials with missing outcome data

**DOI:** 10.1186/s12874-021-01261-6

**Published:** 2021-04-15

**Authors:** Ping-Tee Tan, Suzie Cro, Eleanor Van Vogt, Matyas Szigeti, Victoria R. Cornelius

**Affiliations:** 1 School of Public Health Imperial College London, Medical School Building, St Mary’s Hospital, Norfolk Place, London, UK; 2 Imperial Clinical Trials Unit, Imperial College London, Stadium House, 68 Wood Lane, London, UK

**Keywords:** Controlled multiple imputation, Randomised controlled trials, Missing data, Sensitivity analysis, Multiple imputation

## Abstract

**Background:**

Missing data are common in randomised controlled trials (RCTs) and can bias results if not handled appropriately. A statistically valid analysis under the primary missing-data assumptions should be conducted, followed by sensitivity analysis under alternative justified assumptions to assess the robustness of results. Controlled Multiple Imputation (MI) procedures, including delta-based and reference-based approaches, have been developed for analysis under missing-not-at-random assumptions. However, it is unclear how often these methods are used, how they are reported, and what their impact is on trial results. This review evaluates the current use and reporting of MI and controlled MI in RCTs.

**Methods:**

A targeted review of phase II-IV RCTs (non-cluster randomised) published in two leading general medical journals (The Lancet and New England Journal of Medicine) between January 2014 and December 2019 using MI. Data was extracted on imputation methods, analysis status, and reporting of results. Results of primary and sensitivity analyses for trials using controlled MI analyses were compared.

**Results:**

A total of 118 RCTs (9% of published RCTs) used some form of MI. MI under missing-at-random was used in 110 trials; this was for primary analysis in 43/118 (36%), and in sensitivity analysis for 70/118 (59%) (3 used in both). Sixteen studies performed controlled MI (1.3% of published RCTs), either with a delta-based (*n* = 9) or reference-based approach (*n* = 7). Controlled MI was mostly used in sensitivity analysis (*n* = 14/16). Two trials used controlled MI for primary analysis, including one reporting no sensitivity analysis whilst the other reported similar results without imputation. Of the 14 trials using controlled MI in sensitivity analysis, 12 yielded comparable results to the primary analysis whereas 2 demonstrated contradicting results. Only 5/110 (5%) trials using missing-at-random MI and 5/16 (31%) trials using controlled MI reported complete details on MI methods.

**Conclusions:**

Controlled MI enabled the impact of accessible contextually relevant missing data assumptions to be examined on trial results. The use of controlled MI is increasing but is still infrequent and poorly reported where used. There is a need for improved reporting on the implementation of MI analyses and choice of controlled MI parameters.

**Supplementary Information:**

The online version contains supplementary material available at 10.1186/s12874-021-01261-6.

## Background

Randomised controlled trials (RCTs) are the “gold standard” study design for the evaluation of new and existing medical treatments [1, 2]. However, most RCTs will have some amount of missing data, for example due to participant withdrawal or loss to follow-up. Missing data can compromise the credibility of the trial conclusions, especially when the amount is substantial [3]. This is because any method of statistical analysis requires an untestable assumption about the distribution of the missing data. Analysis should be undertaken under justified primary missing data assumptions using an appropriate statistical approach. The aim is to obtain an unbiased estimate that also accounts for the uncertainty due to missing data [4]. Sensitivity analysis under alternative plausible missing data assumptions should then be performed to assess the robustness of the trial results. It is also important to conduct sensitivity analysis to address deviations from other assumptions used in the statistical model for the main estimator, however we do not consider this further here.

There are three broad categories of assumptions that can be made for missing data: missing completely at random (MCAR), missing at random (MAR) and missing not at random (MNAR) [3, 5]; see Additional file 2: Table S1 for definitions. No missing data assumptions can be universally recommended, as these will be trial specific. Moreover, critically any missing data assumption is untestable. The most plausible assumption for primary analysis should be selected based on the clinical context and understanding of why missing data has arisen. In RCTs, the strong MCAR assumption is typically unlikely to be valid since treatment and early observed responses may affect drop out. As recommended by the US National Research Council (NRC) Panel on Handling Missing Data in Clinical Trials [6], a MAR assumption may often be most reasonable for primary analysis. Analysis under MAR is performed using the observed data and so appealingly, unlike MNAR, does not require any external information to be combined with the observed trial data. Under MAR the distribution of a participants’ data at the end of the study given their earlier observed data, does not depend on whether the data at the end of the study were observed. Therefore regarding missing data, most often sensitivity analyses based on plausible assumptions that depart from MAR should be performed to assess the robustness of results [3]. Depending on the context at hand, sensitivity analyses under alternative MAR specifications (i.e. assuming missingness is dependent on alternative sets of observed covariates) may also be important.

Valid methods to accommodate missing data under MAR include likelihood-based methods, complete case analysis (provided the analysis model incorporates all observed data associated with both missingness and outcome), inverse probability weighting, Bayesian methods and Multiple Imputation (MI) [4, 6, 7]. For analysis under MNAR, selection models or pattern mixture models fitted using maximum likelihood or within a Bayesian or multiple imputation framework can be employed [8, 9].

Despite a wealth of available statistical methods, past reviews have shown that sensitivity analyses under alternative missing data assumptions are not commonly reported [10, 11]. A large gap between methods research and use of principled methods has been identified and attributed to the accessibility of methods [10].

One practical and accessible method for MNAR analysis which follows the pattern-mixture modelling approach and has recently been gaining attention in the statistical literature is controlled multiple imputation. Data are imputed for analysis multiple times under a specified MNAR distribution that the analyst postulates and ‘controls’. The two principle approaches to controlled MI are (i) delta-based imputation and (ii) reference-based imputation [12]. Delta-based MI involves altering the MAR imputation distribution using a specified numerical sensitivity parameter termed ‘delta’ [4, 8] to explore the impact of a better or worse outcome for the unobserved, relative to that predicted based on the observed data under MAR. Reference-based MI was developed in 2013 specifically for use in an RCT setting [13, 14]. In brief, the parameters of the MAR imputation model from a specified reference group (typically the control group) are borrowed to impute missing data in other groups in the trial in a contextually relevant manner. A few imputation options under the reference-based approach are described further below in the methods section. As with any method of statistical analysis, missing data assumptions within controlled MI analyses are untestable.

As controlled imputation methods provide a systematic, more accessible tool for trial analysis under a variety of MNAR assumption, they are particularly useful for sensitivity analysis. Such methods enable the impact of contextually relevant assumptions, that are readily interpretable to clinical colleagues, to be explored. Additionally, since MI can be implemented using inbuilt MI programs in standard statistical software packages complex model coding can be avoided using MI. But it is unknown how often these methods are used in practice, whether they are adequately reported, and what their impact is on trial inferences. It is important that statistical methods are reported accurately and in sufficient detail for research reproducibility and to ensure readers can understand the assumptions behind the analysis to draw fully informed inferences. To thoroughly assess the robustness of trial results readers also need to view sensitivity results alongside primary results.

Two previous reviews examined the use and reporting of MI in RCTs published up to the year 2013 in two leading general medical journals [11, 15]. They found the use of MI under MAR has increased over time, but poor reporting around the methods. They also found limited use of sensitivity analysis under a MNAR assumption within the MI framework. Only one study that reported performing controlled MI as sensitivity analysis was identified in the review by Rezvan between 2008 to 2013 [11].

Motivated by recent methodological developments and increasing attention on controlled imputation methods since 2013, we undertook a targeted review to evaluate the current use and reporting of MI and controlled MI procedures (delta-based and reference-based approach) in RCTs in leading general medical journals. This review extends the reviews by Mackinnon et al. [15] (articles from earliest searchable date to 2008) and Rezvan et al. [11] (articles from 2008 to 2013) with a focus on the use and reporting of controlled MI procedures to highlight good practise. We also aimed to identify the impacts of controlled imputation methods on trial inferences.

## Methods

### Multiple imputation

Standard MI (under MAR) was originally introduced by Rubin in 1978 and involves three main steps [16, 17]. In step one, an imputation model is specified using the observed data that are related to the missingness and multiple datasets are created. Imputed values are sampled from the observed data distribution within a Bayesian framework, which incorporates random variability in the unknown missing data values and parameter values in the imputation model. Secondly, the substantive analysis model of interest is fitted to each of the imputed datasets. Lastly, the results from each of the imputed dataset analyses are combined using Rubin’s rules. This provides a single overall averaged estimate and associated estimate of variance for inference, incorporating the average within-imputation variation and the between-imputation variation across imputed data sets.

Several different methods may be employed to generate imputations from their predictive distribution within a Bayesian framework in step 1 [7, 18]. With missingness on a single variable an underlying regression model may be used. With missingness across multiple variables with a monotone pattern of missingness a series of conditional regression models may be utilized for imputation in a sequential fashion. Alternatively with multivariate missingness, and any missing data pattern, Multivariate Imputation by Chained Equations (MICE), also referred to as Fully Conditional Specification (FCS), may be used [19, 20]. This involves specifying a series of univariate regression models, appropriate for the data type of the variables being imputed, and a Gibbs type sampling procedure is used to impute missing data values in a cyclical fashion. Or a joint model for the observed and missing data, such as a multivariate normal (MVN) model and an iterative Markov Chain Monte Carlo (MCMC) method may be implemented to draw missing data values [21].

In step 3, for Rubin’s combining rules to provide valid inference, the imputation and analysis model must be congenial [22]. In brief, this means the imputation model used in stage 1 must include all the variables and structure in the analysis model used in stage 2 so that the imputation model makes the same assumptions for the data as the analysis model. Additional variables that are thought to be predictive of missingness and outcome, referred to as auxiliary variables, can also be included in the imputation model; This can help strengthen the validity of the underlying MAR assumption. In such circumstances, provided all variables in the analysis model are also in the imputation model, Rubin’s rules will still provide valid inference and imputations can be more efficient with precision increased [22–24]. If however the imputation model omits one or more variable included in the analysis model then Rubin’s rules will not provide valid inference [22, 24].

### Controlled multiple imputation

MI can also be used to explore departures from MAR, i.e. for analysis under a MNAR assumption. This is referred to as controlled MI and includes delta-based MI and reference-based MI. Data is imputed under an alternative MNAR distribution that reflects a contextually relevant scenario for the unobserved data. The imputed datasets are then analysed as with standard MI. As with any method of MNAR analysis careful thought, both clinical and statistical, is required to justify the assumed (untestable) underlying MNAR model.

Reference-based MI can be used when it is postulated that the participants with unobserved data behaved like participants in a designated reference group. Data are imputed following the distribution observed in a particular reference group in the trial, typically another treatment arm. Reference-based MI is appealing because the difference between the MAR and MNAR distribution is described entirely using available trial parameters, rather than requiring the specification of any explicit numerical parameters for the unobserved data

A number of different reference-based multiple imputation approaches can be constructed including: jump to reference (J2R), last mean carried forward (LMCF), copy increments in reference (CIR) and copy reference (CR) [13]. For instance, J2R imputes missing data assuming participants jump to behave like those in the specified reference group (e.g. either the treatment or control arm) following their last observed time point. Carpenter, Roger and Kenward (2013) proposed a general algorithm for referenced based MI of a longitudinal continuous outcome using an underlying MVN model [13]. Further description of these options (not-exhaustive) are presented in Table 1 for a continuous outcome. These reference based MI options can be conducted in Stata using MIMIX [25] or SAS using ‘the five macros’ or ‘miwithd’ [26, 27]. Full technical details on the construction of the appropriate imputation distributions and covariance structure can be found in Carpenter, Roger, and Kenward (2013) [13].

**Table 1:** Options (not exhaustive) for reference-based and delta-based multiple imputation with a continuous outcome

Imputation option	Handling of missing outcome data
Last mean carried forward (LMCF)	Impute assuming all unobserved participants stayed at the mean value of their respective randomised group after their last observed time point.
Copy reference (CR)	Impute assuming all unobserved participants behaved similarly to the behaviour of the specified reference group for the entire duration of the study.
Copy increments in reference (CIR)	Impute assuming all unobserved participants followed the mean increments observed in specified reference group after their last observed time point.
Jump to reference (J2R)	Impute assuming all unobserved participants jumped to the behaviour of the specified reference group after their last observed time point.
Delta	Impute assuming all unobserved participants having a poorer or better response than those observed, by adding or subtracting a “delta” parameter’ to the expected value of the MAR imputed values. “Delta” can be implemented in all treatment groups, or in only one group, or may vary by treatment group or an alternative specified factor.

Reference-based MI can also be performed for recurrent event data and implemented using a negative binomial model [28, 29] or a piecewise exponential model [30]. For binary and ordinal data, Tang et al. [31] demonstrated approaches using sequential logistic regression or a multivariate probit model. For time-to-event (survival) data, event times are often not observed and censored at participants last follow-up. Analysis typically makes the censoring at random (CAR) assumption that, conditional on observed covariates in the model, the event time process is independent of the censoring time process. This is the analogue of the MAR assumption in the time-to-event setting. As with other data types, MI can be used to impute unobserved event times for CAR data [32–34]. To explore the robustness of inferences for survival data to censoring not at random assumptions (informative or dependent censoring) various non-MI methods that can be viewed as time-to-event representatives of selection models [35–41], or pattern mixture models [42, 43] have been proposed. Recently Atkinson et al. [44] extended reference-based MI for time-to-event data and presented a set of reference-based assumptions specifically for survival data under censoring not at random using a Weibull proportional hazards model. Lu et al. presented reference-based MI using a cox proportional hazards model [45]. Zhao et al. has also proposed a nonparametric reference-based MI procedure where the hazard in a specified control group is used within imputation [46]

An alternative method of controlled MI, termed delta-based MI entails modifying the MAR imputation distribution using a specified numerical ‘delta’ parameter, to make predicted responses better or worse than predicted under MAR. For a continuous outcome, ‘delta’, the offset parameter can represent the difference in the mean response between the observed and unobserved cases. For instance, sensitivity analysis can be conducted to explore the impact of assuming a worse outcome than predicted under MAR for those with unobserved data. It is often likely that participants may have a worse response after withdrawing from a trial in the absence of trial medication. Analysis can be repeated with a series of different ‘delta’, representing an increasingly worse/better outcome for the unobserved. For a binary or time to event outcome ‘delta’ can respectively represent the difference in the (log) odds, or hazard, of response between the observed and unobserved cases [31, 34, 47, 48].

As done under MAR, when controlled MI is used, each imputed data set is analysed using the substantive analysis model of interest. Estimates across the imputed data sets are then combined using Rubin’s rules [16] to give a single multiple imputation estimate and measure of variance. It has been shown that controlled multiple procedures (delta- and reference-based) preserve the proportion of information lost due to missing data under MAR in the analysis when Rubin’s rules are implemented, thus they provide valid information anchored inference with missing data [49].

### Search strategy

To evaluate the current application and reporting of MI and controlled MI in RCTs a targeted review of trials published in The Lancet and New England Journal of Medicine (NEJM) between January 2014 and December 2019 was conducted. These two internationally leading medical journals provide indication of the best practise surrounding the reporting of MI and were selected to allow comparison to previous reviews by Rezvan [11] (2008 to 2013 from NEJM and the The Lancet) and Mackinnon [15] (earliest searchable date to 2008 from NEJM, The Lancet, JAMA and BMJ). Articles were identified using a full-text search for the term “multiple imputation” in each journal’s website, restricted by the time period of interest.

### Inclusion criteria

Phase II to IV RCTs were eligible if they used MI for either the primary or sensitivity analysis of the primary outcome. Cluster-randomised controlled trials were excluded because of differing statistical issues to individually randomised trials.

All associated supplementary materials, protocols, statistical analysis plans (SAPs) and web appendices were also reviewed. Corresponding authors were contacted via email to request a copy of their Statistical Analysis Plan (SAPs) when not available online.

### Study selection

Search results were exported to EndNote software. Titles and abstracts were screened by one reviewer (PT) using the pre-defined inclusion and exclusion criteria specified above. Any uncertainties were screened by a second reviewer (SC) to confirm eligibility status. The full text of the remaining articles were each assessed by two reviewers independently (PT, EVV or MS) with any uncertainties regarding eligibility discussed and resolved with a third reviewer (SC).

### Data extraction

Data was extracted onto a standardised data extraction form in excel. We extracted key trial characteristics and for the primary outcome, the proportion of missing data, the method for handling missing data in the primary and sensitivity analysis, and details on the multiple imputation method in primary/sensitivity analysis including: method of MI, specification of variables in the imputation model, commands/procedures (software) used, number of imputations and whether any diagnostic checks were performed. When a trial had more than one primary outcome, the first reported outcome in the trials methods section was used and when an article reported more than one trial, data were extracted for the first trial.

In the subset of trials using controlled MI, we additionally extracted details on the controlled MI procedure including the type (i.e. delta-based or reference-based), numbers of scenarios explored, method of MI, specification of variables in the imputation model, commands/procedures (software) used, number of imputations, whether any diagnostic checks were performed and presented missing data assumptions. If the type of imputation was not explicitly stated, the method used was inferred from the reported software package or references cited when possible. Results of controlled MI analysis and analyses under alternative assumptions were also extracted where relevant.

Data from all RCTs were extracted independently by two researchers (PT, EVV or MS). Any discrepancies, uncertainties or ambiguities experienced during data extraction were discussed and resolved with a third researcher (SC) to reach consensus. The PRISMA guidelines [50] were followed for transparent reporting of systematic reviews (see Additional file 1: Table S1).

### Outcomes

The main outcome measures were (i) the number of trials using multiple imputation; (ii) the number of trials using controlled multiple imputation; (iii) the analysis status of multiple imputation analysis (primary or sensitivity) and (iv) the analysis status of controlled multiple imputation analysis (primary or sensitivity).

For trials using controlled multiple imputation, secondary outcomes included, (i) the number of trials using delta-based MI, (ii) the number of trials using reference based MI, (iii) the differences between results in controlled MI analyses and other performed analyses for the primary outcome. We also assessed the reported details on MI methods for trials using a MI or controlled MI analysis including, specification of the imputation method and imputation model. As this was a review of the use of multiple imputation in randomised trials we did not undertake a risk of bias evaluation.

### Statistical methods

Descriptive statistics were calculated for the trial characteristics and our outcomes of interest. Results of primary and sensitivity analyses for trials using controlled MI analyses were compared by assessing the differences between the treatment effect point estimate, confidence interval (CI) and *p*-value (whether or not the *p* value switched from *p* < 0.05 to *p* > 0.05 or vice versa). We also assessed whether the authors commented on any differences or similarities in the results.

## Results

### Study selection

**Fig. 1:**
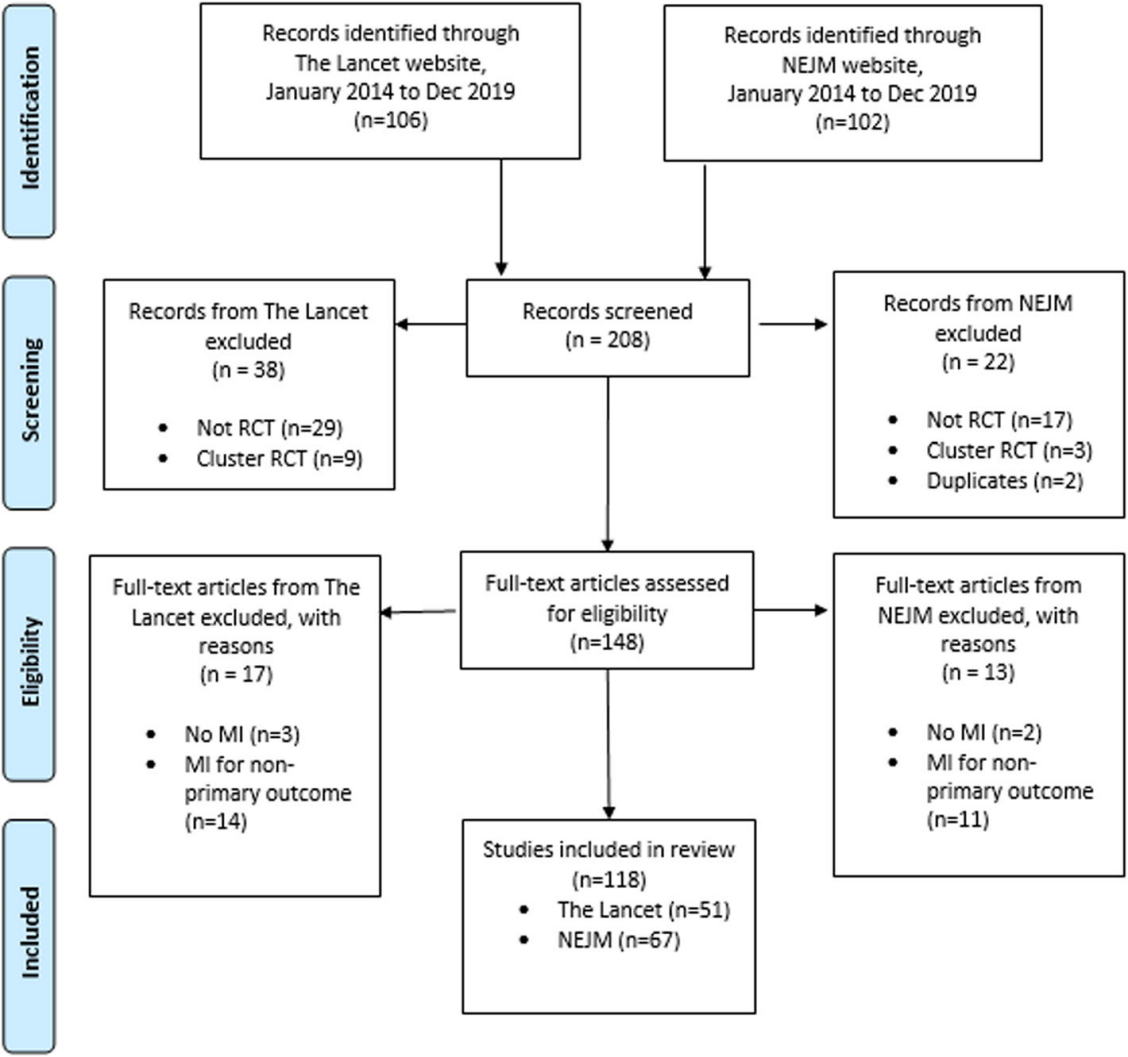
PRISMA flow diagram

The search identified 208 records containing the text “multiple imputation” (MI) between January 2014 and December 2019. After screening abstracts and titles, 148 articles were assessed as potentially eligible after excluding cohort studies, cross-sectional studies, population survey and cluster RCTs. Following full text review, 118 eligible studies were included once studies that had not actually used MI for one or more analysis of the primary outcome were excluded, see Fig. 1.

### Study characteristics

**Fig. 2:**
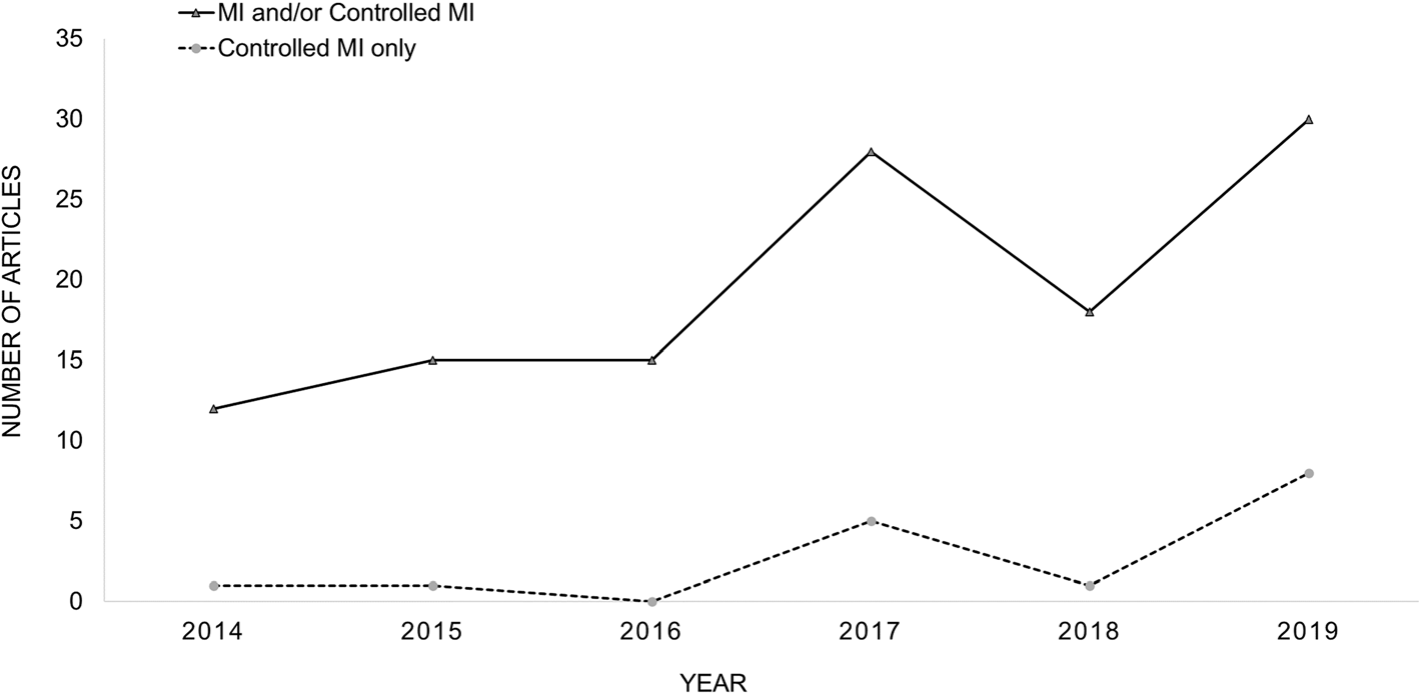
Articles with multiple imputation or controlled multiple imputation in Lancet and NEJM (2014 to 2019)

Figure 2 shows the number of articles using MI under MAR and/or controlled MI in both journals across the 6 years. There were 67 studies in NEJM and 51 studies in the Lancet that included the use of MI and/or controlled MI, which encompassed 9% from a total of 1267 RCTs (phase II-IV, non-cluster) published in both journals over the period, see Fig. 2. The majority of the included RCTs evaluated drug interventions (56%) followed by surgical interventions (19%) with trial sample size ranging from 37 to 20,066 participants, median 592 participants. Table 2 shows the key characteristics of the included studies.

**Table 2:** Key characteristics of included RCTs and methods for handling missing data

	The Lancet (***n*** = 51)		NEJM (***n*** = 67)		Total (***n*** = 118)	
**Type of Intervention**						
Behaviour (diet, exercise, cognitive)	5	10%	4	6%	9	8%
Medical device	6	12%	1	1%	7	6%
Diagnostic	2	4%	0	0%	2	2%
Drug	19	37%	47	70%	66	56%
Health service strategies	5	10%	2	3%	7	6%
Psychological	5	10%	0	0%	5	4%
Surgical	9	18%	13	19%	22	19%
**Number of Participants**						
< 100	2	4%	4	6%	6	5%
100 to 499	24	47%	20	30%	44	37%
500 to 999	16	31%	21	31%	37	31%
1000 to 4999	8	16%	14	21%	22	19%
≥ 5000	1	2%	8	12%	9	8%
**Proportion of Missing Primary Outcome Data**						
< 10%	21	41%	33	49%	54	46%
10 to 19%	17	33%	23	34%	40	34%
20 to 29%	6	12%	4	6%	10	8%
≥ 30%	3	6%	4	6%	7	6%
not clear	4	8%	3	4%	7	6%
**Type of Primary Outcome**						
Binary	20	39%	34	51%	54	46%
Continuous	27	53%	30	45%	57	48%
Count	4	8%	0	0%	4	3%
Time-to-event	0	0%	3	4%	3	3%
**Assessed Differences Between Participants with Complete and Incomplete Data**						
Yes	5	10%	6	9%	11	9%
No	46	90%	61	91%	107	91%
**Method of Handling Missing Data in Primary Analysis**						
MAR MI^a^	22	44%	21	31%	43	36%
Controlled MI	1	2%	1	1%	2	2%
Complete case	19	38%	39	57%	58	49%
Single imputation	5	10%	5	7%	10	8%
Last observation carried forward	3	6%	2	3%	5	4%
**Conducted Missing Data Sensitivity Analysis**						
Yes	36	71%	59	88%	95	81%
No	15	29%	8	12%	23	19%
**Method of Handling Missing Data in Sensitivity Analysis Where Sensitivity Was Conducted**^b^						
MAR MI	28	68%	42	54%	70	59%
Controlled MI	3	7%	11	14%	14	12%
Complete case	6	15%	10	13%	16	13%
Single imputation	1	2%	8	10%	9	8%
Last observation carried forward	1	2%	6	8%	7	6%
Others	2	5%	1	1%	3	3%
**Method of Controlled MI (primary or sensitivity analysis)**						
Reference-based	2	40%	5	45%	7	44%
Delta-based	3	60%	6	55%	9	56%

Data presented as n and %. ^a^One trial using MI under MAR used a hybrid of MI under MAR and worst observation carried forward (single imputation)

^b^23 trials using two or more different statistical method for sensitivity analysis

Percentages are rounded to 0 decimal places so may not sum exactly to 100%

### Reporting and extent of missing data

The proportion of missing primary outcome data was not clear for 7 trials (6%). (Table 2) Excluding trials which had unclear reporting, the level of missingness ranged from 0.1 to 38%, with a median of 10%. Half of the RCTs (*n* = 57, 48%) had 10% or more primary outcome data missing. The primary outcomes imputed were most commonly binary (*n* = 54, 46%) and continuous (*n* = 57, 48%). Four papers (3%) imputed count outcomes while three (3%) papers imputed time-to-event outcomes. Out of the 118 reviewed RCTs, only 11 trials (9%) provided a comparison of participants baseline characteristics by those with observed versus unobserved data.

### Use of standard MI and/or controlled MI and analysis status

Across primary and sensitivity analysis, MI under MAR was used in 110/118 trials. A total of 16/118 studies performed controlled MI (14%). This corresponds to use of MI for one or more analysis of the primary outcome in 9% (110/1267) of all eligible RCTs published in both journals over the period, and controlled MI in 1% (16/1267).

The most widely used methods to handle missing data in the primary analysis were complete case analysis (*n* = 58, 49%) and standard MI under a MAR assumption (*n* = 43, 36%). Two trials (2%) used controlled MI under MNAR in their primary analysis. The other trials used single imputation (*n* = 10, 8%) or last observation carried forward (LOCF) (*n* = 5, 4%) as their primary analysis.

Sensitivity analysis to address the impact of missing data was performed in 95/118 (81%) trials. This included 70/95 (74%) trials using one statistical method for sensitivity analysis, 23/95 (24%) trials using two or more different methods and for 2/95 (2%) trials the methods used for sensitivity analysis were not reported. MAR MI (*n* = 70/95, 74%) was more commonly used in sensitivity analysis, compared to controlled MI under a MNAR assumption (*n* = 14/95, 15%). Other methods used for sensitivity analysis included, complete case analysis (*n* = 16/95, 17%), LOCF (*n* = 7/95, 7%), single imputation (*n* = 9/95, 9%), a mean score approach (using the rctmiss command in Stata [51]) (*n* = 1/95, 1%), baseline observation carried forward (BOCF) (*n* = 1/95, 1%), and Maximum Likelihood (ML) analysis with an EM algorithm (*n* = 1/95, 1%).

### Reporting of MI under MAR

Of the 110 trials using MI under MAR the method for generating imputations was reported for 63 (57%) trials and was most frequently MICE (see Table 3). Of the trials using MICE only 12 provided further detail on the specific types of imputation models used within the MICE procedure (see Table S2). Seven trials indicated regression based MI imputation was performed. Of these, only 4 reported the specific type of regression model utilised; This included one trial with a time-to-event outcome (survival) which used a logistic model for imputation (see Table S3).

**Table 3:** Reporting of methods for MI under MAR

	n (***N*** = 110)	%
**Type of primary outcome**		
Binary	52	47%
Continuous	52	47%
Count	4	4%
Time-to-event	2	1%
**Method of MI**		
Not stated	47	43%
Multiple Imputation using Chained Equations (MICE/FCS)	35	32%
Specified Imputation Model Type(s) within MICE/FCS	12	11%
MCMC MI/algorithm/method	8	7%
Regression based MI	7	6%
Specified imputation Model Type within regression based MI	4	4%
PMM	5	5%
MVN imputation	4	4%
MVN imputation (non-monotone missing patterns) and regression MI model (monotone patterns)	1	1%
MICE (non-monotone missing patterns) and regression MI model (monotone patterns)	1	1%
MCMC (non-monotone missing patterns) and PMM (monotone patterns)	1	1%
Propensity score MI	1	1%
**Specified variables in imputation model**	**52**	**47%**
Imputation model incl. All variables in analysis model only	6	5%
Imputation model incl. All variables in analysis model + auxiliary variables	37	34%
Imputation model did not include all variables in analysis model^a^	9	8%
**Did not specify variables in imputation model**	**58**	**53%**
Imputation model incl. All variables in analysis model + auxiliary variables	8	7%
**Reported the number of multiple imputations**	**70**	**64%**
**No. of imputations**		
5	9	8%
10	8	7%
11–20	25	23%
21–50	17	15%
100	8	7%
200	1	1%
1000	2	2%
Not stated	40	36%
**Specific procedure/command(s) (software) for implementing MI**	**26**	**24%**
IVEware software	1	1%
MICE (R)	3	3%
Proc MI (SAS)	3	3%
Proc MI and Proc MIANALYZE (SAS)	5	5%
Proc MIANALYZE (SAS)	2	2%
Realcom Impute	1	1%
Ice (Stata)	2	2%
MICE (Stata)	1	1%
Mi impute (Stata)	5	%
MI impute and mi estimate (Stata)	1	1%
Missing data module in SPSS 24^b^	2	2%
Not stated	84	76%
**Rubin’s rules used for inference**		
Yes^c^	25	23%
No^d^	1	1%
Not stated	84	76%
**Analysis status**		
Primary	2	13%
Sensitivity	14	87%
**Performed diagnostic check of imputations**	1	1%

^**a**^9 trials did not include all variables in the analysis model in the imputation model and included auxiliary variables. ^**b**^One trial specified that the Multiple Imputation-Automatic method was used. ^**c**^ Explicitly stated (*n* = 18) or inferable from specified software or reference (*n* = 7). ^**d**^One trial reported presented the overall 95% confidence using the mean of the values for the lower and upper confidence intervals. Percentages are rounded to 0 decimal places so may not sum exactly to 100%

The variables included in the imputation model were specified for 52 (47%) trials. All the variables in the analysis model were included in the imputation model for 51 trials (46%), including 6 (5%) trials that specified only the variables in the analysis model in the imputation model, 37 trials (34%) that specified additional auxiliary variables in the imputation model and 8 trials (7%) that did not specify the exact variables included in the imputation model but indicated that the imputation model included all variables included in the analysis model plus additional (not stated) auxiliary variables. Seventy trials (64%) reported the number of imputations, with a median of 20 (IQR 14 to 50, minimum 5, maximum 1000).

For 25 (23%) trials it was explicitly stated, or could be inferred from stated software/references that Rubin’s rules were used to combine estimates across imputed data sets. Only one trial reported a diagnostic check of imputations, consisting of a visual check to compare the distribution of observed and imputed values.

Across the individual extracted fields, 5 (5%) trials provided complete details on the method of MI (i.e. general method of MI specified and exact type of model(s) utilised), variables included in the imputation model, number of imputations and method for combing results post-imputation.

### Reporting of controlled MI

A total of 16 RCTs performed controlled MI under MNAR for primary analysis (*n* = 2) or sensitivity analysis (*n* = 14). This included trials which had continuous (*n* = 9), binary (*n* = 4) and time-to-event (*n* = 3) outcomes, and a proportion of missing data ranging from 1 to 36.4%, median 13% (see Table 4 and Additional file 2: Table S2). Nine trials used delta-based MI and seven trials used reference-based MI. Of the two trials that used controlled MI in primary analysis, one used jump to reference MI, whilst the other used delta-based MI. Of the 14 trials that used controlled MI in sensitivity analysis, 8 used delta-based MI and 6 used some form of reference-based MI.

**Table 4:** Reporting of methods for controlled MI

Controlled MI feature	n (***N*** = 16ª)	%
**Type of primary outcome**		
Binary	4	25%
Continuous	9	56%
Time-to-event	3	19%
**Type of controlled MI**		
Delta-based MI	9	56%
Reference based MI	7	44%
**Method of delta-based MI (*N*** **= 9)**		
MICE^e^	2	22%
MVN imputation (non-monotone missing patterns) and regression MI model (monotone patterns)	1	11%
Kaplan-Meier MI (KMMI)	2^b^	22%
ANCOVA MI	1	11%
Cox model MI	1^†^	11%
Not stated	3	27%
**Method of reference-based MI (*N*** **= 7)**		
MCMC or random draws from a normal distribution with mean equal to subject’s own baseline value^f^	1	14%
Linear MMRM	2	29%
Kaplan-Meier MI (KMMI)	1	14%
Not stated	3	43%
**Specified variables in imputation model**	9	56%
Imputation model incl. All variables in analysis model only	5	31%
Imputation model incl. All variables in analysis model + auxiliary variables	3	19%
Imputation model did not include all variables in analysis model^g^	1	6%
**Did not specify variables in imputation model**	7	44%
Imputation model incl. All variables in analysis model + auxiliary variables	2	13%
**Reported the number of imputations**	13	81%
**No. of imputations**		
5	2	13%
20	1	6%
100	6	38%
1000	4	25%
Not stated	3	19%
**Specific procedure/command(s) (software) for implementing MI**		
Proc MI and Proc MIANALYZE (SAS)	2	13%
Proc MIXED and Proc MIANALYZE (SAS)	2	13%
Not stated	12	75%
**Rubin’s rules used for inference**		
Yes^h^	9	56%
No^c^	1	6%
Not stated	6	38%
**Analysis status**		
Primary	2	13%
Sensitivity	14	87%
**No. scenarios used in sensitivity analysis**^d^	**Median**	**Range**
Median (range)	3	(1–48)
**Performed diagnostic check of imputations**	**0**	**0%**

ª Denominator for variables 16 unless otherwise indicated. ^b^One trial used both KKMI and Cox model MI in two separate sensitivity analyses. ^c^One trial reported using a modified version of Rubin’s rules, “the overall average estimated event rate difference and average estimated variance” (did not incorporate any between imputation variability in the variance calculation). ^d^*N* = 13. Not clear for 1/14 trials using controlled MI in sensitivity analysis. ^e^No further details available on types of models utilised within MICE. ^f^Missing data during the on-treatment period were imputed “using the MI SAS procedure (using Markov Chain Monte Carlo)” and values missing values during the post-treatment period were “multiply imputed using random draws from a normal distribution where the mean was equal to subject’s own baseline value.” ^g^One trial did not include all variables in analysis model and included auxiliary variables in the imputation model. ^h^ Explicitly stated (*n* = 5) or inferable from specified software or reference (*n* = 4). Percentages are rounded to 0 decimal places so may not sum exactly to 100%

The description of the controlled MI procedure provided in the articles and supplementary materials varied in detail between the trials (see Table 4 and Additional file 2, Table S4), with most reporting the information in supplementary materials, and not in the main text. The method of MI model used was not stated in 6/16 (37%) trials using controlled MI. The most common methods of delta-based MI were MICE (*n* = 2) or Kaplan-Meier MI (*n* = 2). None of the two trials using MICE provided further detail on the types of imputation models used within the MICE procedure. Reference-based MI was performed using a linear MMRM (*n* = 2), Kaplan-Meier MI (*n* = 1), or one trial specified using MCMC MI for data missing during the ‘on-treatment’ period or as random draws from a normal distribution with mean equal to subject’s own baseline value for data missing post treatment.

The number of imputations used was reported in 13/16 trials using controlled MI, with a wide range from 5 to 1000 imputations, median 100 (IQR 100 to 1000). Justification for the number of imputations implemented was rarely reported. Only two studies justified 100 imputations to limit to < 1% loss of power compared to full information maximum likelihood, and to generate stable results. Only 4 trials reported specific commands/procedures for implementing controlled MI, but overall 14 articles stated the software they used to conduct their analysis, with SAS being the most common software (*n* = 13) and one use of Stata (*n* = 1). Nine trials (56%) used Rubin’s combining rules for inference, including four trials using reference-based MI and five using delta-based MI. One trial (6%) using reference-based MI used a modification of Rubin’s rules which did not incorporate any between imputation variance in the variance estimate and for 6 (38%) it was not stated or could not be inferred how imputation estimates were combined.

All trials that performed delta-based imputation (*n* = 9) provided some description of their ‘delta’ parameter (see Additional file 2, Table S4 for trial descriptions). The delta parameter was either applied to both treatment and control arms (*n* = 6), or applied only to the treatment arm (*n* = 3). Only 2 trials provided some justification of their ‘delta’ parameter. One trial which used delta-based MI in the primary analysis had a non-inferiority design and used the non-inferiority margin of 0.4% as the “delta” for the treatment arm only and justified that this was to minimise the potential bias towards equivalence. The other trial systematically varied a range of “delta” value to generate 48 imputation scenarios in their sensitivity analysis.

Seven trials performed reference-based imputation (see Additional file 2, Table S4 for trial descriptions). Missing values were imputed to follow the behaviour of participants in the control arm (*n* = 4), the treatment arm (*n* = 2), or the participant’s baseline (*n* = 1).

When controlled MI was used in sensitivity analysis, the number of controlled MI scenarios (i.e. different missing data assumptions) could be inferred for most of the trials (*n* = 13/14). The number of scenarios ranged from 1 to 48 scenarios with a median of 3.

### Comparison of primary and sensitivity results for trials using controlled MI

Of the two trials using controlled MI for primary analysis, one reported no sensitivity analysis whilst the other conducted analysis under MAR as sensitivity analysis (using a Mixed Model for Repeated Measures); The results of the sensitivity analysis were not directly reported, however the trial stated estimated outcomes with imputation were similar without imputation (see Additional file 2, Table S4).

For the 14 trials using controlled MI in sensitivity analysis there was inconsistency in the presentation of results (see Additional file 2: Table S5). Seven trials fully presented the estimate and CI or *p*-value from the primary analysis, and for each of the sensitivity scenarios explored. Others reported a range of estimates or *p*-values as a summary for the sensitivity scenarios (*n* = 5) conducted, or disclosed only the value of the upper bound CI (*n* = 1). One study did not compare the primary and sensitivity results, or comment on the robustness of the primary results.

A comparison of primary and sensitivity results (full or summary) in trials that performed delta-based imputation was possible for 8 trials. Seven trials yielded comparable results to the primary analysis whereas for 1 trial results from the analysis with delta-based imputation (3 out of 4 scenarios showed *p* < 0.05) contradicted the primary result (*p* > 0.05).

In trials that performed reference-based imputation where a comparison of primary and sensitivity results was possible (*n* = 6), 5 studies demonstrated the conclusion of the primary results was robust to the missing data assumptions. For the other study, while the primary result was significant (*p* < 0.05), the result with reference-based imputation showed no difference in treatment effect (*p* > 0.05).

## Discussion

### Use of MI and controlled MI

This targeted review revealed the use of MI and controlled MI has increased from pre-2014 to 2019. The number of RCTs using MI or controlled MI to handle missing data in the primary outcome (118) has increased substantially from a previous review by Rezvan et al. [11] that found 69 RCTs using MI, under MAR, over the previous 6 years study period (2008 to 2013). Collectively with another earlier review by Mackinnon, it is encouraging to observe this continued growing trend of MI which enables principled analysis with missing data. MI under MAR is not a new method, established since 1978 [17]. Given increasing MI research and discussions [8, 33, 52–54], and how MI is now included in many commercial statistical packages, it is not surprising that many researchers are more familiar and confident to use MI.

This is the first targeted review that focussed on the use and reporting of controlled MI (both delta-based and reference-based approach). Sixteen RCTs performed controlled MI as part of their primary outcome analysis. Although the application of controlled MI has increased compared to the one study found to utilise this method in the previous review by Rezvan et al., it is still infrequently used.

Two trials used controlled MI for the primary analysis of the primary outcome. This included a non-inferiority trial using a delta-based approach [55], and a RCT which utilized jump to reference MI where the reference arm was the control arm [56].

It was encouraging to find 81% of RCTs included missing data sensitivity analysis. However, recommendations put forth by regulatory bodies in 2010 to conduct primary analysis, followed by a set of sensitivity analyses under alternative assumptions (e.g. MNAR) [6, 57], has not yet translated to application in the majority of examined RCTs. Many trialists still conduct CCA under MCAR/MAR as their primary analysis and presume MI, under MAR, serves sufficiently as their sensitivity analysis. If the multiple imputation model includes the same variables as the complete case analysis then these analyses will not actually be assessing trial results under differing missing data assumptions. The proportion of RCTs employing MI in the primary analysis has not grown much since the previous review [11].

The findings in this study continue to reveal a translation gap between statistical research of controlled MI and application in RCTs [10, 13, 14]. However, unlike MI, currently, some controlled MI procedures have only recently been available in user-written commercial statistical software programs. This may be a contributing factor to the current low application of controlled MI identified in RCTs.

### Inadequate reporting of MAR MI methods

Despite literature highlighting the importance of reporting imputation methods for research replicability [15, 52], this review found reporting of MI analyses under MAR remains poor. Only 5% trials reported complete details on the implementation of MI under MAR (i.e. specified complete details on method and model(s) used for generating imputations, specified variables in imputation model, number of imputations, and how results were combined post MI).

A large proportion of trials did not include any information on the method of generating imputations. For the trials that did there was most often incomplete information provided with specification of a general method e.g. MICE or MCMC with no further description of the types of models being used within these procedures (e.g. linear/logistic regression etc.)

The majority of trials did not specify the variables included in the imputation model. The standard MI procedure typically uses Rubin’s rules to combine estimated across imputed data sets for inference, which requires all variables included in the analysis model to be included within the imputation model. Due to poor reporting on the variables included in the imputation models and on the use of Rubin’s rules, for most trials there was not adequate information available to assess whether valid inference had been obtained post-MI.

A large proportion of trials (36%) did not describe the number of imputations used nor stated any justification. Whilst 5 to 10 imputations are sufficient for inference, due to Monte Carlo error, more may be required to provide a good level of precision [8, 12]. It has been recommended that the number of imputed data sets be at least as large as the percentage of missing data [53]. One hundred or more imputations may be required to ensure accurate, stable point estimates and standard errors [8]. Just over a third of trials used 20 or less imputations and did not report justification of the number of imputations rendering it hard to establish the accuracy of MI results. Monte carol standard errors were not reported in any reviewed trials. Only one trial reported checking the distribution of MAR imputed values again observed values, making it generally impossible to infer anything further about the predictive quality of imputations. These poor reporting results are similar to the findings of earlier reviews and indicate that little progress has been made [11].

### Inadequate reporting of controlled MI methods

Reporting of controlled MI analyses was poor. Only 5/16 trials using controlled MI reported complete details on the implementation of controlled MI. The majority of trials implementing controlled MI did not fully describe the method or model(s) used to generate imputations, and just under half (44%) did not specify the variables included in the imputation model.

There has been some debate in the statistical literature about the appropriate variance estimator to use when implementing controlled MI, and in particular when reference based assumptions are made [58]. This is because the data generating mechanism is deliberately inconsistent with the assumption of the analysis model to some degree (i.e. uncongenial). In the MAR setting, the usual MI variance estimator, Rubin’s variance estimator, requires congeniality. Controlled MI procedures as described here, including both reference and delta-based, use an MAR model to build a MNAR distribution for imputation. Rubin’s variance estimate, has been shown to provide information anchored inference when such MI procedures are used [49]. That is, the proportion of information lost due to missing data under MAR is approximately preserved in the analysis. The underlying MAR model that provides the building blocks for the MNAR imputation model should therefore be congenial with the analysis model i.e. contain all the variables and structure (including interaction terms). Otherwise, if the underlying MAR model is uncongenial with the analysis model there will be an additional source of uncongeniality in the controlled MI analysis affecting the MI inference. In contrast, the usual empirical long-run sampling variance of the treatment estimator can exhibit entirely different behaviour; within reference based analyses, as a consequence of borrowing information between treatment arms, it undesirably decreases as the proportion of missing data increases. Several researchers have proposed alternative analytical methods for variance estimation when reference based assumptions are made that target the empirical long-run sampling variance [59, 60]. Of the trials performing controlled MI, 38% did not state or provide software/references which would enable one to infer how they combined their results across multiply imputed data sets for inference. Since different variance estimates can result in quite different inference it is important that trialists clearly report how estimates have been combined across imputed data sets. The combination of poor reporting on the variables included in the imputation models and on the use of Rubin’s rules, means for most trials using controlled MI there was not adequate information available to assess whether valid inference had been obtained.

In trials using controlled MI for sensitivity analysis, there was a large range in the reported number of controlled MI scenarios (1 to 48 scenarios) assessed. There are many potential ways a RCT can structure sensitivity scenarios, which may depend on its intervention, clinical context, setting and hypotheses of the impact of missing data [14]. Careful clinical and statistical thought should justify any MNAR model due to its untestable nature. Sensitivity analyses should be comprehensive to cover sufficient plausible assumptions of missing data, and are unbiased to the experimental treatment [6, 57].

The RCTs that performed delta-based approaches demonstrated how the approach can be applied in many ways. “Delta” was added onto imputed missing values in one treatment arm, or in both arms, to make the estimated unobserved outcomes either better or worse. However, justification on the selected arm or the delta values were not provided for all trials. Authors should provide this information to ensure the readers understand the underlying missing data assumptions to inform inferences from the results.

The reference-based approach has multiple options (e.g. J2R, LMCF, CIR, CR). For trials that performed a reference-based imputation approach, none provided a rationale for their selected approach. Researchers should explain why the selected imputation approach is appropriate for their context [14]*.*

### Impact of controlled MI on the robustness of the primary trial result

This targeted review is unique because we also assessed the impact of controlled MI on trial results for RCTs that performed a controlled MI and non-controlled MI analysis. There was inconsistency in the presentation of results; some trials provided full results from their primary analysis and each of the imputed scenarios, while others selectively reported sensitivity results.

Most trials that performed delta-based or reference-based imputation in sensitivity analysis demonstrated results were robust to different missing data assumptions. However two trials, one using delta-based imputation and another using reference-based imputation demonstrated contradicting results versus primary analyses, i.e. *p* value switched from *p* < 0.05 to *p* > 0.05 or vice versa. This illustrates how incorporating MNAR missing data assumptions can change the inference of the trial. However, the contradicting results were not highlighted or explained in the trials.

### Limitations

This review was limited to RCTs using MI from the Lancet and NEJM to allow comparisons to previous reviews by Revzan [11] and Mackinnon [15]. The use and extent of reporting of MI and/or controlled MI in these two journals is unlikely to be generalisable to articles published in other journals. RCTs published in these two high impact journals will have detailed statistical review encouraging better handling of missing data, as well as more detailed reporting. Therefore, this study may provide an overestimate of the use and reporting standards of MI and controlled MI in other journals. As only 16 cases of controlled MI were identified, our review of reporting standards for these analyses is further limited. We focussed on the handling of missing data, including missing data assumptions. Naturally in any statistical analysis, other underling modelling assumption are also important for trialsist to assess and justify (e.g. proportional hazards when utilising a Cox model), however this was not addressed in this review. As only RCTs using MI were included the review does not provide any information on the broader use of missing data sensitivity analyses or assumptions within RCTs. Initial screening of titles and abstracts from the search results in this review was performed by one reviewer. However, a second reviewer assessed any uncertainties at this initial stage and the full text of articles reviewed to made a final judgment on eligibility were reviewed by two independent reviewers which is considered an acceptable method [61].

### Future recommendations

It is important for trialists to incorporate sensitivity analysis under alternative missing data assumptions and present results alongside the primary result to prevent selective reporting. We acknowledge it is often difficult to include all these details in the main text because many journals have strict word limits. However, authors can still include this information in their supplementary materials or web appendices. It is not definitive that results from all scenarios of sensitivity analysis must be aligned with the primary result to be considered robust [57]. Rather, it is case dependent, and if sensitivity analysis gives a different conclusion, the researcher should attempt to understand why and report the possible explanation [52].

Future research should focus on establishing a consensus on the details required to be reported for a trial that performs MI or controlled MI. The development of reporting guidelines for the use of multiple imputation in randomisation trials that use robust and widely accepted methods, such as the Delphi consensus method, are required [62, 63]. Clear reporting is essential to ensure reproducibility and ensure clinician and policy makers fully understand the underlying assumptions and draw appropriate inferences.

Reflecting on the practice observed from this review and guidance proposed by others [52], for RCTs that perform any MI analysis for the primary outcome the following should be clearly reported in the article: i) fully describe the method and model(s) used to generate imputations (ii) specify all the variables included in the imputation model iii) report the number of imputations, iv) report the software package and procedures/commands used, v) report how results are combined across imputed data sets for inference (e.g. Rubin’s rules) vi) provide results as the pooled point estimate, CI interval and *p*-value. When specifying the variables included in the imputation model, as described by Sterne et al., it may also be necessary to describe how any non-normally distributed variables and categorical variables were dealt with [52]. When controlled MI is implemented, in addition to the above 6 aspects, the following should also be clearly reported vii) describe the controlled MI scenario(s) used and state the underlying assumption(s), viii) describe the rationale for choosing the scenario(s), ix) provide results of each controlled MI analysis as pooled point estimate, CI interval and p-value and x) provide possible explanations if results support contrary inferences to that of other conducted analyses where relevant.

## Conclusions

This review demonstrates the growing use of MI in RCTs. While the use of controlled MI has also increased compared to the previous review, it is still infrequent and when used poorly reported. Sensitivity analysis under alternative missing data assumptions using principled methods such as Controlled MI should be more widely adopted to test the robustness of trial results. Careful clinical and statistical thought should justify any MNAR model. When MI or controlled MI is used, imputation methods should be completely reported, and encompass the aforementioned recommendations. Collectively with efforts from editors and peer reviewers to demand the proper use and reporting of missing data handling, clinicians and policy-makers will then be able to make more informed conclusions about the validity of trial results.

## Supplementary Information

**Additional file 1 Table S1.** PRISMA checklist for reporting systematic reviews.

**Additional file 2: Table S1.** Underlying assumptions for missing data. **Table S2.** Model details for trials implementing MICE (*n*=12). **Table S3.**Model details for trials implementing regression based MI (*n*=4). **Table S4.** Key characteristic of the 16 RCTs that performed controlled MI. **Table S5.** Assessing the robustness of the results from the 16 RCTs that performed controlled MI.

## Data Availability

The datasets used and/or analysed during the current study are available from the corresponding author on reasonable request.

## Abbreviations

ANOVA: Analysis of variance
CCA: Complete case analysis
CI: Confidence interval
CIR: Copy increments in reference
CR: Copy reference
J2R: Jump to reference
LMCF: Last mean carried forward
MAR: Missing at random
MCAR: Missing completely at random
MI: Multiple Imputation
MICE: Multiple Imputation by Chained Equations
MNAR: Missing not at random
MVN: Multivariate normal
QoL: Quality of life
RCT: Randomised controlled trial
