# Supplementary material for: A review of the use of controlled multiple imputation in randomised controlled trials with missing outcome data
Source: BMC Med Res Methodol. 2021 Apr 15;21:72. doi: 10.1186/s12874-021-01261-6 (PMC8048273; doi:10.1186/s12874-021-01261-6)
Supplement: Supplementary file 2 — Additional file 2: Table S1. Underlying assumptions for missing data. Table S2. Model details for trials implementing MICE (n=12). Table S3.Model details for trials implementing regression based MI (n=4). Table S4. Key characteristic of the 16 RCTs that performed controlled MI. Table S5. Assessing the robustness of the results from the 16 RCTs that performed controlled MI. [file 12874_2021_1261_MOESM2_ESM.docx]

| **Table S1 Underlying assumptions for missing data** | |
| --- | --- |
| **Assumptions** | **Definition** |
| Missing completely at random (MCAR) | MCAR assumes the missing data are unrelated to observed and unobserved data. When outcomes are MCAR, the distribution of the unobserved outcomes will be no different than for those observed in the trial. |
| Missing at random (MAR) | MAR assumes the missing data are related to observed data, but not the unobserved data. Under MAR, the unobserved outcomes can be modelled from those who remain in the trial with similar characteristics and in longitudinal settings, similar profile over time until missing. |
| Missing not at random (MNAR) | MNAR assumes missing data are dependent on the unobserved outcomes in some way. When data are MNAR, the missing values cannot be modelled exclusively from the data of the observed participants |

**Table S2 – Model details for trials implementing MICE (n=12)**

| **Models utilised within MICE/FCS** | **N** |
| --- | --- |
| PMM | 1 |
| ordinal, linear and logistic regression models utilised | 1 |
| linear models for continuous variables and logistic or multinomial regression models for categorical variables | 2 |
| logistic regression | 3 |
| logistic, poisson and linear regression | 1 |
| continuous variables were imputed using linear regression and binary variables were imputed using logisticregression | 2 |
| logistic regresion (primary) and PMM | 1 |
| Ordinal logistic model | 1 |

**Table S3 – Model details for trials implementing regression based MI (n=4)**

| **Type of primary outcome** | **Type of regression based MI model** | **N** |
| --- | --- | --- |
| Binary | Logistic | 1 |
| Time to event | Cox (proportional hazard) | 1 |
| Time to event | Weibull | 1 |
| Time to event | Logistic | 1 |

| **Table S4 Key characteristic of the 16 RCTs that performed controlled MI**. | | | | | | | |
| --- | --- | --- | --- | --- | --- | --- | --- |
| **Reference** | **Type of Intervention** | **N randomised** | **Proportion of Missing Primary Outcome Data** | **Type of Primary Outcome** | **MI under MAR**  **(Analysis status /Type of MI)** | **Controlled MI**  **(Analysis status/ No. of scenarios and description)** | **No. of imputation*/Software** |
| **Delta-based imputation** | | | | | | | |
| Hagen 2017, Lancet | exercise | 414 | 17.6% | continuous | Sensitivity /not stated | Sensitivity/ 6 scenarios: Missing participants assumed to have worse outcomes (delta-parameter of one point higher) or better outcomes (one point lower) in both treatment and control arms, and also in each arm only. “Missing data were assumed to be better than expected (one point lower than the imputed value assuming missing at random, and similarly one point worse than expected). We also restricted these assumptions of being better and worse to each group individually.” | not stated/ Stata 13 |
| Robert 2019, Lancet | surgical | 253 | 22.5% | continuous | Primary/ not stated | Sensitivity/ 1 scenario: Missing participants assumed to have the addition of delta-parameter of 7% to the imputed values in the treatment group. “with multiple imputation and addition of 7% to the imputed values in the OAGB group” | 5 imputations/ SAS 9.4 |
| Berlowitz 2017, NEJM | drug | 9361 | 13.0% | continuous | Sensitivity/ A two step procedure involving, MVN imputation for non-monotone missing data patterns followed by a series of linear regression models for monotone patterns | Sensitivity/ 4 scenarios: Missing participants in the intensive treatment group were imputed (as under MAR using MVN MI and a series of linear regression models) with delta-parameter of 1 to 4 points. “We evaluated sensitivity to the MAR assumption by deterministically shifting the imputed PCS and MCS scores for participants in the intensive-treatment group” | 100 imputations/ SAS 9.4 – Proc mi and proc mi analyze). |
| Collins 2017, NEJM | diet | 1273 | 4.4% | binary | Primary/ MICE using Rubins’ rules. Imputation model included all variables in analysis model plus additional auxiliary variabels (not listed) | Sensitivity/ 6 scenarios: Data was imputed as under MAR (using MICE). The odds of outcome event were assumed to be between half and twice as high in infants with missing data compared to children with observed data, with differences applied to one or both treatment and control arms. | 100 imputations/ SAS 9.3 |
| Saver 2017, NEJM | surgical | 980 | 23.9% | time to event | Sensitivity/ Proportional hazards multiple  imputation (PHMI) method described by Zhao et. al.(2016)[45] | Sensitivity/ 48 scenarios: The conditional cumulative incidence function for events during missing years of observations was varied using a group-specific HR of having an event, theta (hazard of premature discontinuation relative to the hazard of remaining in the trial). Theta(treatment) and theta(control) ranged from 1 to 2.5, with increment of 0.25, using KMMI method described by Zhao et. al.(2014)[46]. “a multiple imputation analysis with systematic variation of the hazard ratio for patients who withdrew from the trial versus patients who continued in the trial.” | 100 imputations/ SAS 9.3 |
| Makrides 2019, NEJM | drug | 5544 | 1% | binary | Primary/ MICE using Rubin’s rules. Imputation model uncluded all variables within the analysis model and auxiliary variables. | Sensitivity/ 6 scenarios: Following MICE, the odds of outcome event were assumed to be between half and twice as high in women with missing data compared to women with observed data, with differences applied to one or both treatment groups. | 100 imputations/ SAS 9.4 |
| Pratley 2019, Lancet | drug | 711 | 5% | continuous | no standard MI | Primary/ 1 scenario: The imputation model was an analysis of covariance. Results were combined using Rubins’ rules. A value of 0·4% point (the non-inferiority margin) was added to multiply imputed values at week 26 for the oral semaglutide treatment arm only to minimise the potential bias towards equivalence in the estimation of the treatment policy. “We used a pattern mixture model with multiple imputation to handle missing data at week 26  for the primary endpoint” ”We based  both the imputation and the analysis on ANCOVA models. We combined the results using Rubin’s rule” ”Before testing for non-inferiority versus subcutaneous liraglutide, we added a value of 0.4% (the non-inferiority  margin) to imputed values at week 26 for the oral semaglutide group” | 1000 imputations/ SAS 9.4M2 |
| Yamamura 2019, NEJM | drug | 83 | 37% | Survival | no standard MI | Sensitivity/ 4 scenarios: To evaluate the influence of early censoring, four post hoc analyses with the use of MI for patients with censored data, excluding patients who were still continuing in the trial at the data cutoff date were conducted. Kaplan Meier MI and Cox model MI.  In Model 1, MI with a Kaplan–Meier model was applied on the basis of Hsu and Taylor (2009)[50] with 100 times iteration; in Model 2, MI with a Cox proportional-hazards model was applied on the basis of Jackson et. al. (2014)[26] with 100 times iteration; in Model 3, MI with a Kaplan–Meier model was applied on the basis of Lipkovich et. al. (2016)[23] with 100 times iteration; and in Model 4, MI with a Cox proportional-hazards model was applied on the basis of Lipkovich et. al. (2016)[23] with 100 times iteration. | 100 imputations/ not stated |
| Butler 2019, NEJM | health service strategies | 653 | 17% | binary | Sensitivity/not stated | Sensitivity/ 12 scenarios: Sensitivity analyses exploring missing data mechanisms assumed data MNAR and comprised of PMMs for the analysis of the Clinical COPD Questionnaire (CCQ) total score outcome, and scenario-based analysis for the antibiotic consumption outcome. A series PMMs with mean in unobserved outcome minus mean in observed outcome ranging from 0 (MAR assumption) to 4 (MNAR assumption) was included in the analyses. | 20 imputations/ not stated |
| **Reference-based imputation** | | | | | | | |
| O’Neil 2018, Lancet | drug | 957 | 6.8% | continuous | no standard MI | Primary/ 1 scenario: Jump to reference MI, where missing values in the active treatment arm were imputed from participants randomly assigned to the placebo arm. “using a jump-to-reference multiple imputation (J2R-MI)  approach based on 1000 iterations of the dataset.” Rubins’ combination rules used post-imputation. | 1000 imputations/ SAS 9.4 |
| Wesson 2019, Lancet | drug | 217 | 4.1% | binary | no standard MI | Sensitivity/ 1 scenario: Imputed using copy reference-control approach. “The missing data from participants in both treatment groups who discontinued early were constructed from the observed data in the placebo group” | not stated/ SAS 9.4 |
| Distler 2019, NEJM | drug | 576 | 13.5% | continuous | no standard MI | Sensitivity/ 3 scenarios (described below)/ MI via a linear MMRM including same variables as analysis model using PROC MIXED and PROC MIANALYZE SAS 9.2 / 1000 imputations  Imputed using varied reference-based procedures for missing participants, based on status of alive or dead at assessment timepoint:  Scenario 1, the missing endpoint for those alive were assumed to have a similar rate of decline as in participants from the corresponding treatment group who prematurely discontinued the trial drug but had an observed endpoint. Missing participants who died were assumed to have a similar rate of decline as in control placebo participants with an observed value who prematurely discontinued the trial drug with the most severe decline.  Scenario 2, the missing endpoint for those alive was assumed to have a similar rate of decline as in participants from the placebo control group who prematurely discontinued the trial drug but had an observed endpoint. Missing participants who died were assumed a similar rate of decline as in control placebo participants with an observed value who prematurely discontinued the trial drug with the most severe decline.  Scenario 3, the missing endpoint for those alive was assumed to have a similar rate of decline as all placebo participants who were included in the primary analysis. Missing participants who died was assumed to have a similar rate of decline as all control placebo participants included in the primary analysis with the most severe declines. | 1000 imputations/ SAS 9.2, PROC MIXED and PROC MIANALYZE |
| Goadsby 2017, NEJM | drug | 955 | 36.4% | continuous | Sensitivity/ not stated | Sensitivity/ not clear: Imputed with an “assumption of MNAR (control-based pattern imputation)” for continuous endpoints. | not stated/ SAS 9.3 |
| Mack 2019, NEJM | surgical | 1000 | 1.6% | time to event | Sensitivity / KMMI – inference was based on “the overall average estimated event rate difference and average estimated variance”, method described by Zhao et. al.(2014)[46] | Sensitivity/ 1 scenario: Imputed with KMMI and reference-based (control) approach, where missing data (did not have a primary endpoint event and did not complete the follow up) for the treatment arm was assumed to be similar to the control surgical arm (who either had primary endpoint events or completed the 1 year follow up). MAR assumed for those in control arm. Inference was based on “the overall average estimated event rate difference and average estimated variance.” “All TAVR subjects from the original ITT Population who did not have a primary endpoint event and did not complete 1 year follow up have been imputed based on …  informative missing assumption (i.e,. they  behaved as the observed Surgery subjects who either had primary endpoint events or  completed the 1 year follow up).” | 5 imputations/ SAS |
| Richeldi 2014, NEJM | drug | 513 | 15.0% | continuous | no standard MI (Primary analysis performed using a MMRM) | Sensitivity/ 3 scenarios: Imputed using a linear MMRM using PROC MIXED and PROC MIANALYZE SAS 9.2 including same variables as analysis model with varied reference-based procedures for missing participants, based on the status of alive or dead at the assessment timepoint. Rubin’s rules used:  Scenario 1, the missing endpoint for those alive was assumed to be at a similar rate of decline as in participants from the corresponding treatment group who prematurely discontinued the trial drug but had an observed endpoint. Missing participants who died were assumed a similar rate of decline as in the control placebo participants with an observed value who prematurely discontinued the trial drug with the most severe declines.  Scenario 2, the missing endpoint for those alive were assumed to be at a similar rate of decline as in participants from the placebo control group who prematurely discontinued the trial drug but had an observed endpoint. Missing participants who died were assumed to have a similar rate of decline similar to the control placebo participants with an observed value who prematurely discontinued the trial drug with the most severe declines.  Scenario 3, the missing endpoint for those alive was assumed to have a similar rate of decline as all placebo participants who were included in the primary analysis. Missing participants who died were assumed to have a similar rate of decline as all control placebo participants included in the primary analysis with the most severe declines. | 1000 imputations/ SAS 9.2, PROC MIXED and PROC MIANALYZE |
| Robinson 2015, NEJM | drug | 2341 | 9.2% | continuous | no standard MI | Sensitivity/ 1 scenario: Missing values during the 'on treatment' period were “ multiply imputed using a model assuming “missing at random” using MCMC and MI SAS procedure; “missing calculated LDL cholesterol values during the post-treatment period were multiply imputed using random draws from a normal distribution where the mean was equal to subject’s own baseline value”.  Results combined using Rubin’s formulae (PROC MI ANALYZE) | 100 imputations/ SAS 9.2 – PROC MI and PROC MIANALYZE |
| * Number of imputations are the same for trials using standard MI and controlled MI  Multiple imputation (MI); MI using chained equations (MICE); MI using multivariate normal model (MVN); Kaplan-Meier multiple imputation (KMMI); missing at random (MAR); missing not at random (MNAR); pattern mixture model (PMM) | | | | | | | |

| **Table S5 Assessing the robustness of the results from the 16 RCTs that performed controlled MI.** | | | | |
| --- | --- | --- | --- | --- |
| **Reference** | **Result of CC/ without imputation** | **Result of MAR MI** | **Result of Controlled MI** | **Robustness of Result** |
| **Controlled MI as primary analysis** | | | | |
| Pratley 2019, Lancet | N.A. | N.A. | Oral semaglutide was non-inferior to subcutaneous liraglutide, MD –0·1% (95% CI –0·3 to 0·0); p<0·0001 for non-inferiority; and superior to placebo, MD–1·1%, 95%CI –1·2 to –0·9; p<0·0001. | No sensitivity analysis reported. |
| O’Neil 2018, Lancet | N.A. | N.A. | Mean was –2·3% for placebo vs –6·0% for 0·05 mg, –8·6% for 0·1 mg), –11·6% for 0·2 mg, –11·2% for 0·3 mg, and –13·8% for 0·4 mg for semaglutide groups with all showing significant (p≤0·0055). Mean reductions was ≥0·2 mg for semaglutide vs liraglutide with all all showing significant (–13.8% to –11.2% vs –7.8%). | It was reported that estimated outcomes with imputations were similar to the observed outcome without imputation, due to the high proportion (93%) of on-treatment and off-treatment participants retained in-trial. |
| **Controlled MI as primary analysis** | | | | |
| Hagen 2017, Lancet | MD –1·01 (95% CI −1·70 to −0·33); p=0·004 | MD not reported, 95% CI −1·03 to −0·70 for analysis with imputation | | Controlled MI and MI (both as sensitivity analysis) yielded similar significant conclusion to the primary analysis without missing data imputation, though with smaller 95% CI. |
| Robert 2019, Lancet | MD –3·3% (90% CI –9.1 to 2.6); p=0·0024 | MD upper bound 90% CI of 3.9%; p=0·0066 (full result not reported) | MD upper bound 90% CI 5.9%; p=0·024 (full result not reported) | Although MI and controlled MI (both as sensitivity analysis) yielded similar significant conclusion to the primary analysis without imputation, the p-values for the sensitivity analysis was slightly larger. |
| Berlowitz 2017, NEJM | Mean -0.01(95% CI -0.12 to 0.11); p=0.90 | Mean 0.00 (95% CI -0.12 to 0.11); p=0.95 | Mean 0.07 (95% CI -0.19 to 0.04); p= 0.21, mean 0.15 (95% CI -0.26 to -0.03); p= 0.014, mean -0.21 (95% CI -0.33 to -0.10); p <0.001, and mean -0.28 (95% CI -0.40 to -0.17); p<0.001. | Primary analysis without imputation and MI under MAR showed no significant mean score differences between the treatment and control group. However, three out of the four sensitivity scenarios with controlled MI yielded contradicting conclusions instead, with significant differences favouring the standard treatment (when imputed with shifted values 2, 3 and 4 points). |
| Collins 2017, NEJM | N.A. | RR 1.13 (95% CI 1.02 to 1.25); p=0.02 | RR 95% CI 1.12 to 1.13 (full result and p-value for all scenarios were not reported). | It was reported that all sensitivity analysis with controlled MI produced similar conclusions compared to the primary analysis with standard MI. The sensitivity analysis produced tighter 95% CI. |
| Saver 2017, NEJM | HR 0.55 (95% CI 0.31 to 0.999); p=0.046 | HR 0.50 (95% CI 0.28 to 0.89); p=0.02 | All sensitivity analysis produced point estimate HR in favour of the treatment device, but with varied level of significance. In 42 scenarios: HR 0.33 to 0,57; p=0.0003 to 0.04. In 6 scenarios: HR 0.33 to 0,57; p=0.053 to 0.16. | Results of sensitivity analyses with MI were consistent with those of the primary analysis without imputation. All controlled MI scenarios resulted in estimates favouring the treatment arm, but with varied significance. Nominal statistical significance was lost when the event rate in the treatment group during censored periods was more than 1.5 times as high as the event rate during the observed periods. |
| Makrides 2019, NEJM | N.A. | RR 1.13 (95% CI 0.79 to 1.63); p=0.50 | All sensitivity analyses produced similar treatment effect estimates range of RR = 1.13 to 1.15 (full result and P-value for each scenario was not reported). | All sensitivity analyses produced similar treatment effect estimates to MI, suggesting that findings were robust to assumptions about the missing data. |
| Yamamura 2019, NEJM | HR 0.38 (0.16 to 0.88); p=0.02 | N.A. | Model 1: HR 0.34 (95% CI 0.14 to 0.78); p=0.01. Model 2: HR 0.37 (95% CI 0.16 to 0.86); p=0.03. Model 3: HR 0.44 (95% CI 0.20 to 0.95); p=0.04. Model 4: HR 0.35 (95% CI 0.15 to 0.81); p=0.02. | The sensitivity analysis of time to any relapse, including both protocol-defined and non–protocol-defined relapses, was consistent with the analysis of protocol-defined relapse. |
| Butler 2019, NEJM | OR 0.31 (95% 0.20 to 0.47); p<0.001 | OR 0.33 (95% 0.21 to 0.52); p<0.001 | Sensitivity 1: OR 0.39 (95% CI 0.27 to 0.58); p<0.001. Sensitivity 2: OR 0.39 (95% CI 0.27 to 0.55); p<0.001. Sensitivity 3: OR 0.28 (95% CI 0.19 to 0.41); p<0.001. Sensitivity 4: OR 0.94 (95% CI 0.66 to 1.33); p<0.728. | The results demonstrates that, for all but the most extreme and implausible assumptions (i.e.that missing CRP participants all consumed antibiotics and missing control participants did  not), the conclusions drawn on the CC remain robust to various missing data assumptions. |
| Wesson 2019, Lancet | 37% (95% CI 23% to 49%); p<0.0001 | N.A. | 36% (95% CI 24% to 49%); p<0.0001 | Sensitivity analysis with controlled MI yielded similar results and conclusion to the primary analysis without imputation. |
| Distler 2019, NEJM | MD 40.95 (95% CI 2.9 to 79.0); p=0.04 | N.A. | MD 30.00 (95% CI -6.22 to 66.22; p=0.10), MD 32.93 (95% CI -3.19 to 69.06); p=0.07, MD 33.86 (95% CI -2.03 to 69.75); p=0.06. | Result of sensitivity analysis with controlled MI (p=0.06 to 0.10) was inconsistent with the results of primary analysis without imputations (p=0.04). |
| Goadsby 2017, NEJM | Mean 3.2 days (70 mg), 3.7 days (140 mg) for treatment group, vs. mean 1.8 days for placebo group (p<0.001 for each dose vs. placebo) | not clear | not clear | not clear |
| Mack 2019, NEJM | 8.5% vs 15.1%; AD −6.6% (95%CI −10.8 to −2.5); p<0.001 and HR 0.54 (95% CI 0.37 to 0.79); p=0.001 | 8.5% vs. 15.2%; AD −6.7%, 95%CI −10.7 to −2.7 and HR 0.53, 95% CI 0.37 to 0.78; p-value reported as “significant” | 8.6% vs. 15.2%; AD −6.6% (95%CI −10.6 to −2.6) and HR 0.54 (95% CI 0.37 to 0.78); p-value reported as “significant” | Results of sensitivity analyses with MI and controlled MI were consistent with those of the primary analyses without imputations. |
| Richeldi 2014, NEJM | MD 125.3 (95% CI 77.7 to 172.8) | N.A. | MD 120.3 (95% CI 75.8 to 164.8), MD 114.8 (95% CI 69.9 to 159.7), MD 113.9 (95% CI 69.2 to 156.5) | The estimates of treatment effects (though slightly lower) and the corresponding 95% CI from the sensitivity analysis with controlled MI were consistent with the results of the primary analysis without imputation. |
| Robinson 2015, NEJM | LSMD −61.9±1.3 (95% CI −64.3 to –59.4); p<0.001 | N.A. | LSMD –58.5±1.3 (95%CI –61.1 to –55.8); p<0.001 | Sensitivity analysis with the use of MI yielded conclusions (though with smaller % change from baseline) similar to the CC. |
| Multiple imputation (MI); hazard ratio (HR); relative risk (RR); missing at random (MAR); standard deviation (SD); mean difference (MD); complete case (CC); absolute difference (AD); least square mean difference (LSMD); confidence interval (CI); odds ratio (OD) | | | | |
